# An unruptured, thrombosed 10 cm right coronary artery aneurysm mimicking a pericardial cyst

**DOI:** 10.1186/1749-8090-8-2

**Published:** 2013-01-07

**Authors:** Aakash Chauhan, Harsha Musunuru, Richard L Hallett, Mark Walsh, Szabolcs Szabo, Walter Halloran

**Affiliations:** 1 Indiana University School of Medicine, Indianapolis, IN, USA; 2 Department of Emergency Medicine, Philadelphia College of Osteopathic Medicine, Memorial Hospital, South Bend, IN, USA; 3 Northwest Radiology Network, Indianapolis, IN, USA; 4 Cardiology Associates Inc, South Bend, IN, USA; 5 CardioThoracic Surgery, P C, South Bend, IN, USA; 6 Department of Orthopaedic Surgery, Allegheny General Hospital, 1307 Federal St 2nd Floor, Pittsburgh, PA 15212, USA; 7 7Philadelphia College of Osteopathic Medicine, 4170 City Avenue, Philadelphia, PA 19131, USA

**Keywords:** Giant coronary artery aneurysm, Right coronary artery, Coronary artery bypass graft, CABG

## Abstract

Giant coronary artery aneurysms are a rare and potentially life-threatening condition. A 47 year old male presented with a progressive 6 month history of dyspnea and acute right sided chest pain. During the patients work-up, a 10 cm × 10 cm right coronary artery aneurysm was discovered on CT scan and confirmed by cardiac catheterization. The patient was emergently taken to the operating room for aneurysmal resection with placement of a greater saphenous vein bypass graft. There were no post-operative complications and the etiology of this patient’s aneurysm was determined to be a congenital ectatic dilation of his right coronary artery. The patient is doing well at 2 years of clinical follow-up.

## Background

Giant coronary artery aneurysm’s are a very rare and life-threatening cardiovascular condition. The presentation of a giant coronary artery aneurysm can resemble an acute coronary syndrome with thrombosis of the aneurysmal sac and subsequent ischemic symptoms from compromised blood flow. However other serious complications such as rupture of the sac and direct compressive effects within the thoracic cavity can also cause serious morbidity and even be fatal for some patients. Therefore immediate diagnosis and cardiothoracic surgical intervention is important in decreasing morbidity and mortality for these patients.

## Case report

A 47 year old male presented to the Emergency Department (ED) with right sided pleuritic chest pain for six hours. Several years prior to his presentation, he had sustained a fall from approximately 3 meters, with minor posterior chest wall trauma. The patient’s past medical and social history were unremarkable and he took no medications. On review of systems, the patient reported an increasing level of fatigue and exertional dyspnea over the past seven months. On exam, the patient’s vital signs were stable and cardiac auscultation revealed a pericardial friction rub with no murmurs or gallops. Electrocardiogram (ECG) showed an incomplete right bundle branch block with non-specific T wave changes. The cardiac enzymes revealed a slightly elevated Troponin-I level of 0.10 ng/mL.

**Figure 1:**
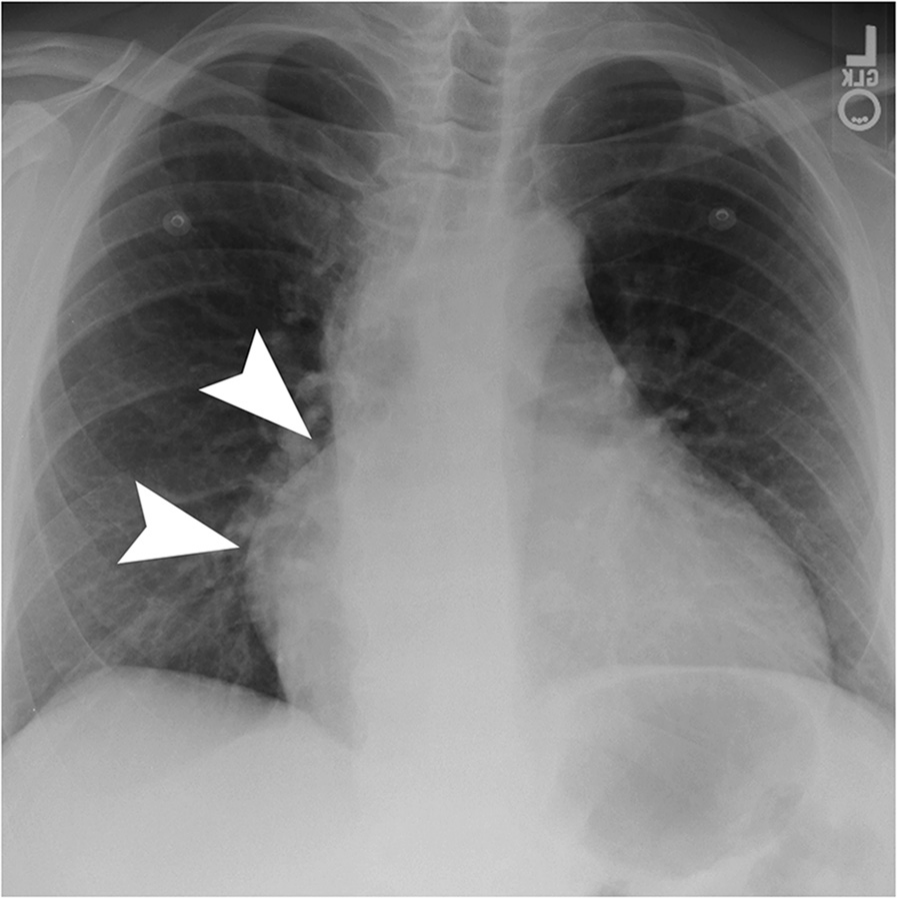
Frontal view of chest demonstrates an abnormal double density along the right heart border (arrowheads).

**Figure 2:**
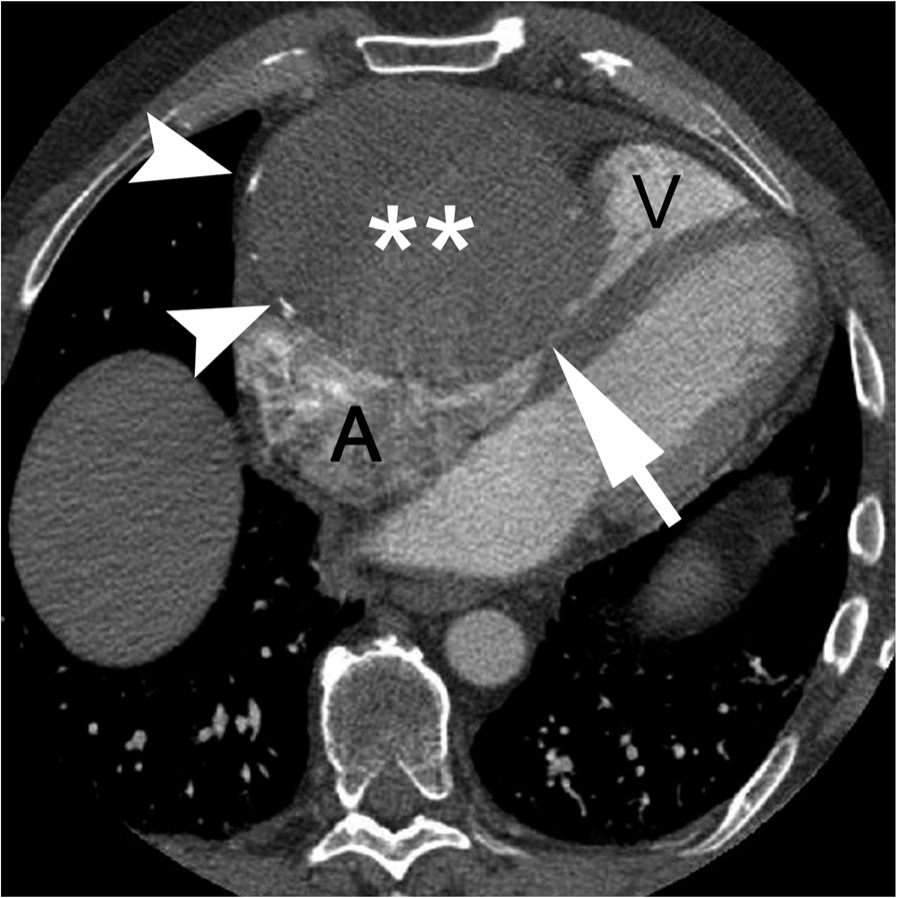
**Axial image from CTA.** There is a large mass (**) with mural calcifications (arrowheads). There is marked compression of the tricuspid valve and mid-heart regions (arrow) between the right atrium (**A**) and right ventricle (**V**).

**Figure 3:**
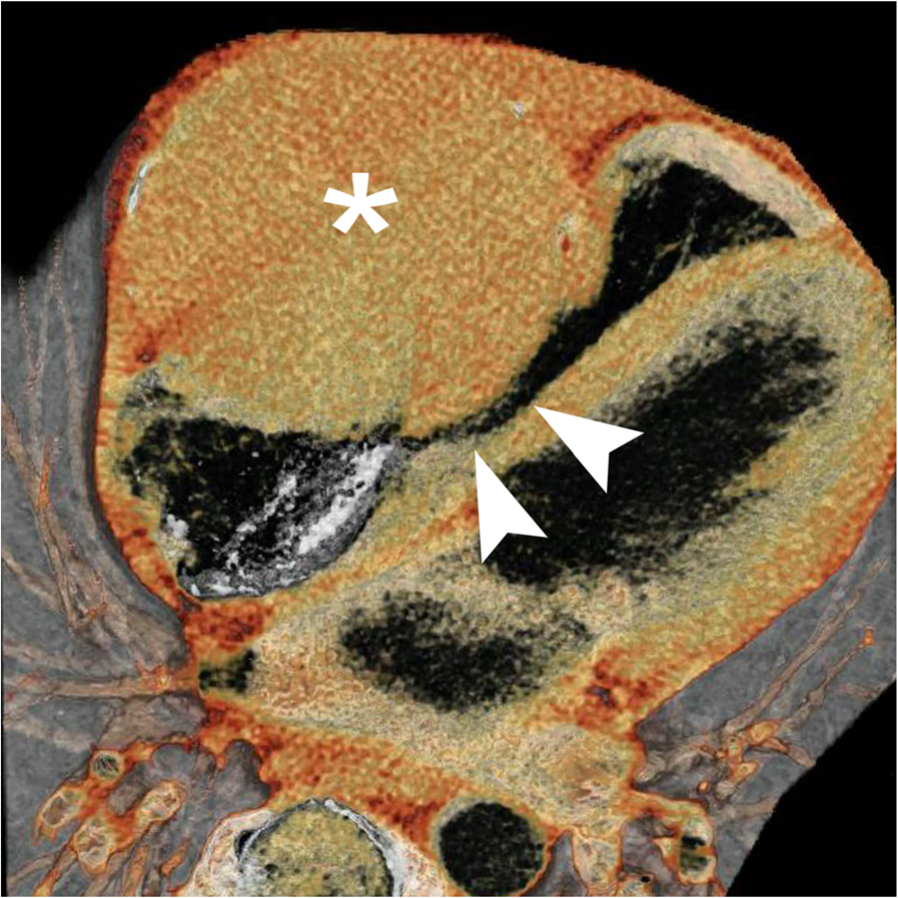
CT Reconstruction of Aneurysm - Blood-pool inversion volume rendered 4-chamber image shows the extent of luminal compression and restriction in diastolic filling of the RV and tricuspid areas (arrowheads) by the mass (*).

**Figure 4:**
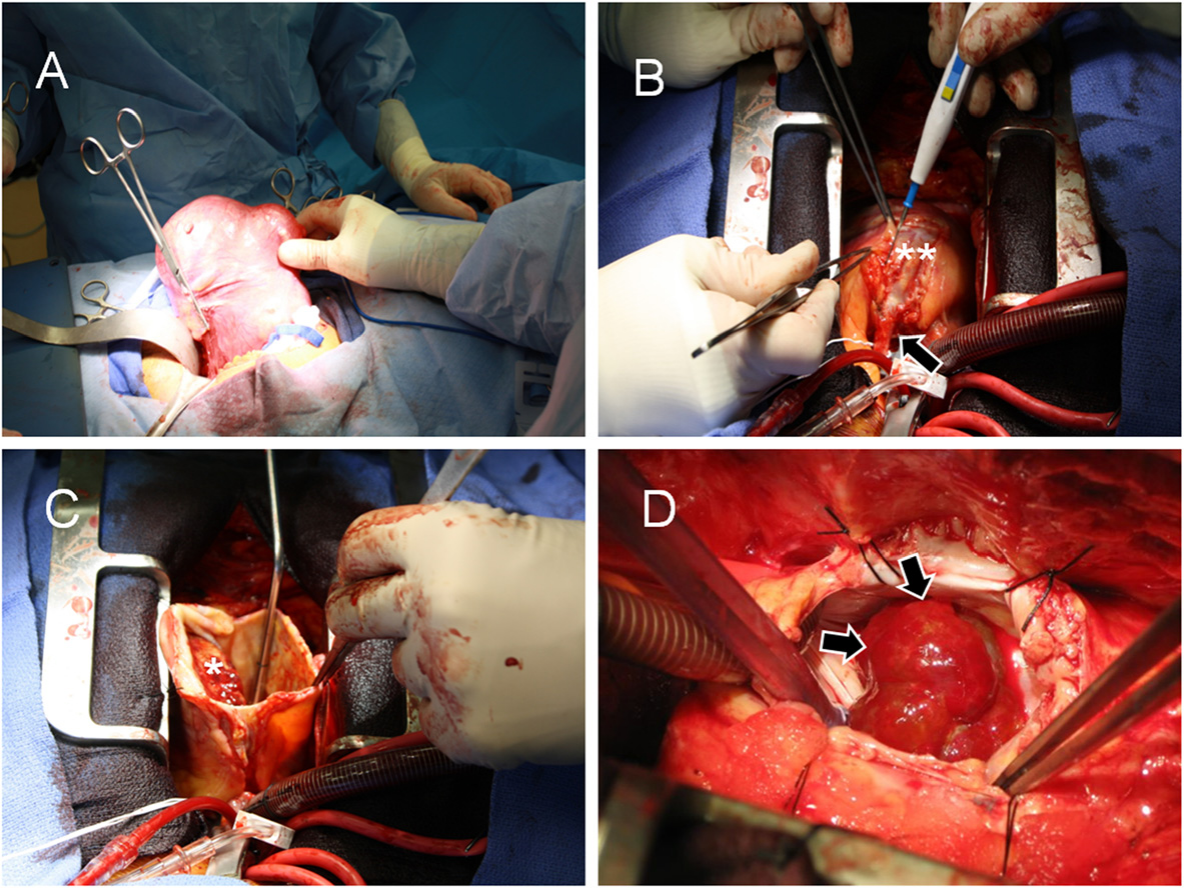
**Intra-operative Images of Giant Right Coronary Artery Aneurysm Resection.** (**A**) Mobilization of aneurysm prior to institution of cardiopulmonary bypass. (**B**) Surgical exposure of the lesion demonstrates a giant aneurysm (**) that is contiguous with the right coronary artery (arrow). (**C**) Opened giant aneurysm with obstructive thrombus (*). (**D**) Intraluminal thrombus (arrows) adherent to the intimal layer of the giant aneurysm.

Initial imaging with chest X-ray (Figure 1) was performed and a contrast enhanced computed tomography (CT) scan of the chest revealed a low attenuation mass located along the right heart anteriorly that measured approximately 10 cm by 10 cm with no significant enhancement (Figure 2). At this point we considered a differential diagnosis of a pericardial cyst versus a true aneurysm or pseudoaneurysm of the right coronary artery (RCA). Echocardiogram revealed a left ventricular ejection fraction of 65-70%, no pericardial effusion, and a well circumscribed echolucent mass compressing the right ventricle (not shown). Selective coronary angiography was performed to further define the abnormality and the RCA injection confirmed the mass to be a giant right coronary artery aneurysm (not shown). CT reconstructions were also performed, which revealed the extent of right heart obstruction by the aneurysm (Figure 3). The patient was taken to the operating room for resection of the lesion under cardiopulmonary bypass using a median sternotomy approach. Intra-operative examination of the lesion revealed a true saccular aneurysm measuring approximately 10 cm by 10 cm that nearly obliterated the right atrial cavity and severely compressed the right ventricle (Figure 4A-D). No other abnormalities were identified and the specimen was sent for histopathology and cultures. Continuity of the RCA was re-established by utilizing a saphenous vein interposition graft. Myocardial function was excellent at the completion of the reconstruction and the patient had an uneventful post-operative recovery without complications. He was seen in late follow-up (2 years) and has returned to full activity. Post-operative CT of the chest reveals the restoration of normal coronary artery anatomy.

## Conclusion

Coronary artery aneurysm’s (CAA) are localized dilations of the artery that exceed the diameter by 1.5 times the largest adjacent normal vessel or a 50% increase in the arterial diameter compared with an adjacent arterial segment [1]. Giant CAA’s are furthermore defined as a general vessel dilation of 2 cm or greater in diameter [2]. A giant CAA is very rare with a reported incidence in a cardiac surgical population of 0.02%, with right coronary arteries affected greater than left [2,3]. The common etiologies behind CAA’s are atherosclerosis, trauma, congenital malformations, infection, vasculitic diseases, and stent placement [1,4]. Giant coronary aneurysms are more likely to be congenital as opposed to smaller coronary artery aneurysms in which the significant majority are atherosclerotic or vasculitic (e.g. Kawasaki’s disease) by origin [3]. If not identified early or managed appropriately, complications such as thrombosis of the vessel with resultant myocardial ischemia, aneurysmal rupture with acute pericardial tamponade, or direct compressive effects from the mass lesion resembling an obstructive cardiomyopathy can occur [5,6].

The histopathology of the aneurysm in our patient showed only chronic perivascular adventitial inflammation and revealed no distinct etiology such as infection or atherosclerosis as the cause. We also considered the traumatic accident the patient suffered seven years prior to be an unlikely cause, since the reported trauma was relatively mild and posteriorly directed, as well no prior evidence found on CT of previous sternal or rib fractures. Our examination, history, and pathologic analysis suggest that this was more likely a congenital ectatic dilatation of his RCA which had progressed over many years. Although his presenting symptoms resembled an acute coronary syndrome due to the thrombosis of the giant aneurysm, his 2-month history of prodromal symptoms was likely due to the chronic compression of his right sided cardiac chambers by the lesion.

## Consent

Written informed consent was obtained from the patient for publication of this Case report and any accompanying images. A copy of the written consent is available for review by the Editor-in-Chief of this journal.

## Competing interests

All of the authors declare that they have no competing interests.

## Authors’ contributions

AC is the primary author, HM and MW assisted in manuscript editing, RLH created imaging and reconstructions, and WH is the senior author and operating surgeon for this case. All authors read and approved the final manuscript.
